# 3-(4-Bromo­phenyl­sulfin­yl)-2,4,6,7-tetra­methyl-1-benzofuran

**DOI:** 10.1107/S1600536812001973

**Published:** 2012-01-21

**Authors:** Hong Dae Choi, Pil Ja Seo, Uk Lee

**Affiliations:** a Department of Chemistry, Dongeui University, San 24 Kaya-dong Busanjin-gu, Busan 614-714, Republic of Korea; b Department of Chemistry, Pukyong National University, 599-1 Daeyeon 3-dong, Nam-gu, Busan 608-737, Republic of Korea

## Abstract

In the title compound, C_18_H_17_BrO_2_S, the 4-bromo­phenyl ring makes a dihedral angle of 89.03 (6)° with the mean plane of the benzofuran fragment. In the crystal, mol­ecules are linked by weak inter­molecular C—H⋯O and C—H⋯π inter­actions.

## Related literature

For the pharmacological activity of benzofuran compounds, see: Aslam *et al.* (2009 ▶); Galal *et al.* (2009 ▶); Khan *et al.* (2005 ▶). For natural products with benzofuran rings, see: Akgul & Anil (2003 ▶); Soekamto *et al.* (2003 ▶). For the crystal structures of related compounds, see: Choi *et al.* (2010**a* ▶,b* ▶).
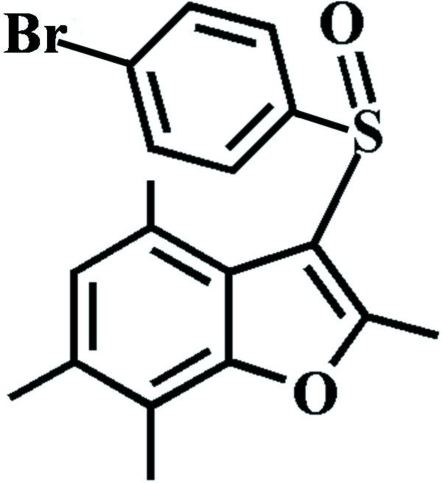


## Experimental

### 

#### Crystal data

C_18_H_17_BrO_2_S
*M* *_r_* = 377.29
Orthorhombic, 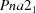

*a* = 12.0900 (4) Å
*b* = 20.8119 (10) Å
*c* = 6.4865 (2) Å
*V* = 1632.11 (11) Å^3^
*Z* = 4
Mo *K*α radiation
μ = 2.65 mm^−1^
*T* = 173 K
0.28 × 0.27 × 0.06 mm

#### Data collection

Bruker SMART APEXII CCD diffractometer
Absorption correction: multi-scan (*SADABS*; Bruker, 2009 ▶) *T*_min_ = 0.524, *T*_max_ = 0.857
8826 measured reflections
3658 independent reflections
2943 reflections with *I* > 2σ(*I*)
*R*_int_ = 0.037

#### Refinement

*R*[*F* ^2^ > 2σ(*F* ^2^)] = 0.034
*wR*(*F* ^2^) = 0.076
*S* = 0.99
3658 reflections
203 parameters
1 restraint
H-atom parameters constrained
Δρ_max_ = 0.29 e Å^−3^
Δρ_min_ = −0.29 e Å^−3^
Absolute structure: Flack (1983 ▶), 1607 Friedel pairs
Flack parameter: 0.005 (8)

### 

Data collection: *APEX2* (Bruker, 2009 ▶); cell refinement: *SAINT* (Bruker, 2009 ▶); data reduction: *SAINT*; program(s) used to solve structure: *SHELXS97* (Sheldrick, 2008 ▶); program(s) used to refine structure: *SHELXL97* (Sheldrick, 2008 ▶); molecular graphics: *ORTEP-3* (Farrugia, 1997 ▶) and *DIAMOND* (Brandenburg, 1998 ▶); software used to prepare material for publication: *SHELXL97*.

## Supplementary Material

Crystal structure: contains datablock(s) global, I. DOI: 10.1107/S1600536812001973/xu5447sup1.cif

Structure factors: contains datablock(s) I. DOI: 10.1107/S1600536812001973/xu5447Isup2.hkl

Supplementary material file. DOI: 10.1107/S1600536812001973/xu5447Isup3.cml

Additional supplementary materials:  crystallographic information; 3D view; checkCIF report

## Figures and Tables

**Table 1:** Hydrogen-bond geometry (Å, °) *Cg*1 and *Cg*2 are centroids of the C2–C7 benzene ring and the C13–C18 bromo­phenyl ring, respectively.

*D*—H⋯*A*	*D*—H	H⋯*A*	*D*⋯*A*	*D*—H⋯*A*
C18—H18⋯O2^i^	0.95	2.58	3.397 (3)	144
C10—H10*B*⋯*Cg*1^ii^	0.98	2.87	3.604 (3)	132
C12—H12*B*⋯*Cg*2^iii^	0.98	2.84	3.671 (3)	143

Symmetry codes: (i) 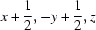; (ii) 
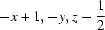
; (iii) 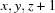.

**Fig. 1.**
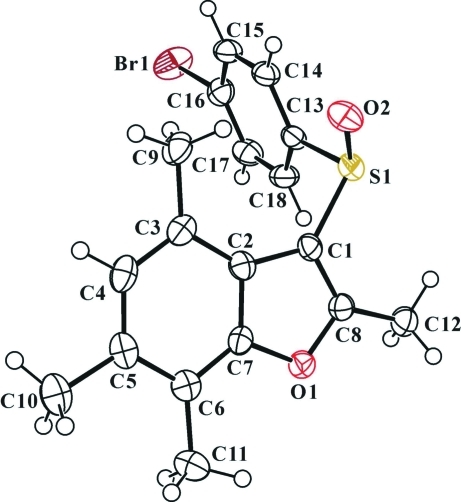
The molecular structure of the title compound with the atom numbering scheme. Displacement ellipsoids are drawn at the 50% probability level. H atoms are presented as small spheres of arbitrary radius.

**Fig. 2.**
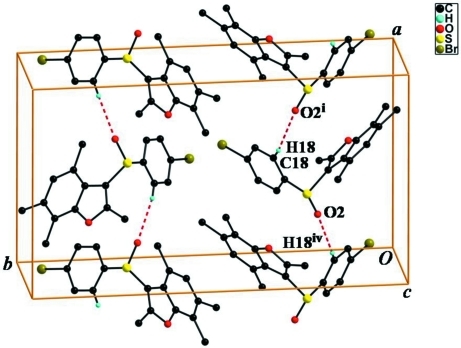
A view of the C—H···O interactions (dotted lines) in the crystal structure of the title compound. H atoms non-participating in hydrogen-bonding were omitted for clarity. [Symmetry codes: (i) x + 1/2, - y + 1/2, z; (iv) x - 1/2, - y + 1/2, z.]

**Fig. 3.**
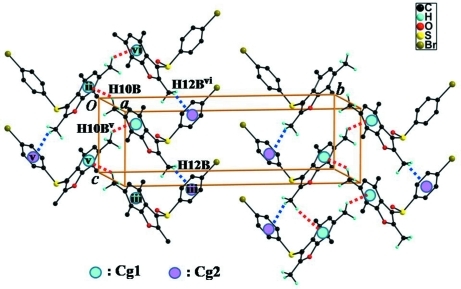
A view of C—H···π interactions (dotted lines) in the crystal structure of the title compound. H atoms non-participating in hydrogen-bonding were omitted for clarity. [Symmetry codes: (ii) - x + 1, - y, z - 1/2; (iii) x, y, z + 1; (v) - x + 1, - y, z + 1/2; (vi)x, y, z - 1.]

## supplementary crystallographic information

### Comment

Benzofuran analogues have drawn much attention owing to their valuable biological properties such as antibacterial and antifungal, antitumor and antiviral, and antimicrobial activities (Aslam *et al.*, 2009, Galal *et al.*, 2009, Khan *et al.*, 2005). These benzofuran derivatives occur in a wide range of natural products (Akgul & Anil, 2003; Soekamto *et al.*, 2003). As a part of our continuing study of 2,4,6,7-tetramethyl-1-benzofuran derivatives containing either 3-(4-fluorophenylsulfinyl) (Choi *et al.*, 2010*a*) or 3-(4-chlorophenylsulfinyl) (Choi *et al.*, 2010*b*) substituents, we report herein the crystal structure of the title compound.

In the title molecule (Fig. 1), the benzofuran unit is essentially planar, with a mean deviation of 0.014 (2) Å from the least-squares plane defined by the nine constituent atoms. The dihedral angle between the 4-bromophenyl ring and the mean plane of the benzofuran fragment is 89.03 (6) °. The crystal packing (Fig. 2) is stabilized by weak intermolecular C—H···O hydrogen bonds (Table 1, first entry). The crystal packing (Fig. 3) is further stabilized by intermolecular C—H···π interactions (Table 1, second & third entry, Cg1 and Cg2 are the centroids of the C2–C7 benzene ring and the C13–C18 4-bromophenyl ring, respectively).

### Experimental

77% 3-chloroperoxybenzoic acid (224 mg, 1.0 mmol) was added in small portions to a stirred solution of 3-(4-bromophenylsulfanyl)-2,4,6,7-tetramethyl-1-benzofuran (325 mg, 0.9 mmol) in dichloromethane (30 mL) at 273 K. After being stirred at room temperature for 4h, the mixture was washed with saturated sodium bicarbonate solution and the organic layer was separated, dried over magnesium sulfate, filtered and concentrated at reduced pressure. The residue was purified by column chromatography (benzene) to afford the title compound as a colorless solid [yield 71%, m.p. 452–453 K; *R*_f_ = 0.46 (benzene)]. Single crystals suitable for X-ray diffraction were prepared by slow evaporation of a solution of the title compound in ethyl acetate at room temperature.

### Refinement

All H atoms were positioned geometrically and refined using a riding model, with C—H = 0.95 Å for aryl and 0.98 Å for methyl H atoms. *U*_iso_(H) = 1.2*U*_eq_(C) for aryl and 1.5*U*_eq_(C) for methyl H atoms.

### Crystal data

**Table:**

C_18_H_17_BrO_2_S	*F*(000) = 768
*M**_r_* = 377.29	*D*_x_ = 1.535 Mg m^−^^3^
Orthorhombic, *P**n**a*2_1_	Mo *K*α radiation, λ = 0.71073 Å
Hall symbol: P 2c -2n	Cell parameters from 3034 reflections
*a* = 12.0900 (4) Å	θ = 2.6–26.5°
*b* = 20.8119 (10) Å	µ = 2.65 mm^−^^1^
*c* = 6.4865 (2) Å	*T* = 173 K
*V* = 1632.11 (11) Å^3^	Block, colourless
*Z* = 4	0.28 × 0.27 × 0.06 mm

### Data collection

**Table:**

Bruker SMART APEXII CCD diffractometer	3658 independent reflections
Radiation source: rotating anode	2943 reflections with *I* > 2σ(*I*)
graphite multilayer	*R*_int_ = 0.037
Detector resolution: 10.0 pixels mm^-1^	θ_max_ = 27.5°, θ_min_ = 2.0°
φ and ω scans	*h* = −15→15
Absorption correction: multi-scan (*SADABS*; Bruker, 2009)	*k* = −14→27
*T*_min_ = 0.524, *T*_max_ = 0.857	*l* = −8→8
8826 measured reflections	

### Refinement

**Table:**

Refinement on *F*^2^	Secondary atom site location: difference Fourier map
Least-squares matrix: full	Hydrogen site location: difference Fourier map
*R*[*F*^2^ > 2σ(*F*^2^)] = 0.034	H-atom parameters constrained
*wR*(*F*^2^) = 0.076	*w* = 1/[σ^2^(*F*_o_^2^) + (0.0182*P*)^2^] where *P* = (*F*_o_^2^ + 2*F*_c_^2^)/3
*S* = 0.99	(Δ/σ)_max_ = 0.001
3658 reflections	Δρ_max_ = 0.29 e Å^−^^3^
203 parameters	Δρ_min_ = −0.29 e Å^−^^3^
1 restraint	Absolute structure: Flack (1983), 1607 Friedel pairs
Primary atom site location: structure-invariant direct methods	Flack parameter: 0.005 (8)

### Special details

**Table:**

Geometry. All esds (except the esd in the dihedral angle between two l.s. planes) are estimated using the full covariance matrix. The cell esds are taken into account individually in the estimation of esds in distances, angles and torsion angles; correlations between esds in cell parameters are only used when they are defined by crystal symmetry. An approximate (isotropic) treatment of cell esds is used for estimating esds involving l.s. planes.
Refinement. Refinement of F^2^ against ALL reflections. The weighted R-factor wR and goodness of fit S are based on F^2^, conventional R-factors R are based on F, with F set to zero for negative F^2^. The threshold expression of F^2^ > 2sigma(F^2^) is used only for calculating R-factors(gt) etc. and is not relevant to the choice of reflections for refinement. R-factors based on F^2^ are statistically about twice as large as those based on F, and R- factors based on ALL data will be even larger.

### Fractional atomic coordinates and isotropic or equivalent isotropic displacement parameters (Å^2^)

**Table:**

	*x*	*y*	*z*	*U*_iso_*/*U*_eq_	
Br1	0.48579 (2)	0.445878 (18)	−0.20739 (7)	0.05430 (12)	
S1	0.36475 (5)	0.25057 (4)	0.52550 (12)	0.03236 (16)	
O1	0.62687 (13)	0.14452 (9)	0.6057 (3)	0.0293 (4)	
O2	0.25673 (14)	0.22004 (11)	0.4737 (3)	0.0426 (5)	
C1	0.47176 (18)	0.19373 (14)	0.5032 (4)	0.0265 (6)	
C2	0.49653 (18)	0.14497 (14)	0.3496 (4)	0.0271 (6)	
C3	0.4518 (2)	0.12236 (13)	0.1632 (4)	0.0296 (6)	
C4	0.5086 (2)	0.07291 (16)	0.0671 (5)	0.0341 (7)	
H4	0.4800	0.0571	−0.0597	0.041*	
C5	0.6055 (2)	0.04454 (13)	0.1445 (4)	0.0321 (6)	
C6	0.6495 (2)	0.06553 (13)	0.3332 (4)	0.0302 (6)	
C7	0.5935 (2)	0.11587 (13)	0.4230 (4)	0.0263 (6)	
C8	0.5520 (2)	0.19176 (13)	0.6489 (4)	0.0275 (6)	
C9	0.3473 (2)	0.14958 (16)	0.0704 (4)	0.0373 (7)	
H9A	0.3632	0.1916	0.0091	0.056*	
H9B	0.2912	0.1544	0.1783	0.056*	
H9C	0.3198	0.1204	−0.0364	0.056*	
C10	0.6606 (2)	−0.00863 (15)	0.0254 (5)	0.0460 (8)	
H10A	0.7390	0.0017	0.0058	0.069*	
H10B	0.6247	−0.0132	−0.1093	0.069*	
H10C	0.6539	−0.0490	0.1021	0.069*	
C11	0.7512 (2)	0.03784 (15)	0.4304 (5)	0.0401 (7)	
H11A	0.7537	0.0499	0.5763	0.060*	
H11B	0.8169	0.0546	0.3601	0.060*	
H11C	0.7496	−0.0091	0.4183	0.060*	
C12	0.5771 (2)	0.23122 (14)	0.8323 (4)	0.0341 (6)	
H12A	0.5872	0.2032	0.9521	0.051*	
H12B	0.5157	0.2609	0.8583	0.051*	
H12C	0.6450	0.2558	0.8081	0.051*	
C13	0.40119 (18)	0.30180 (12)	0.3141 (4)	0.0280 (6)	
C14	0.32284 (19)	0.31628 (13)	0.1674 (4)	0.0304 (6)	
H14	0.2519	0.2968	0.1740	0.036*	
C15	0.3471 (2)	0.35925 (14)	0.0098 (5)	0.0325 (6)	
H15	0.2940	0.3689	−0.0936	0.039*	
C16	0.4504 (2)	0.38754 (13)	0.0075 (4)	0.0336 (6)	
C17	0.5294 (2)	0.37388 (14)	0.1551 (5)	0.0351 (7)	
H17	0.5999	0.3939	0.1497	0.042*	
C18	0.50500 (18)	0.33121 (14)	0.3091 (6)	0.0328 (6)	
H18	0.5584	0.3217	0.4121	0.039*	

### Atomic displacement parameters (Å^2^)

**Table:**

	*U*^11^	*U*^22^	*U*^33^	*U*^12^	*U*^13^	*U*^23^
Br1	0.04980 (18)	0.0588 (2)	0.0543 (2)	−0.01458 (15)	−0.0108 (2)	0.0187 (2)
S1	0.0254 (3)	0.0424 (4)	0.0292 (3)	0.0041 (3)	0.0027 (3)	−0.0035 (3)
O1	0.0267 (8)	0.0342 (11)	0.0269 (9)	0.0003 (8)	−0.0012 (8)	−0.0024 (8)
O2	0.0204 (8)	0.0585 (15)	0.0490 (13)	−0.0016 (8)	0.0056 (8)	0.0010 (11)
C1	0.0225 (11)	0.0324 (16)	0.0246 (13)	−0.0003 (10)	0.0043 (11)	−0.0007 (11)
C2	0.0235 (12)	0.0294 (17)	0.0283 (15)	−0.0036 (10)	0.0051 (10)	0.0010 (11)
C3	0.0284 (12)	0.0340 (17)	0.0264 (13)	−0.0081 (11)	−0.0011 (11)	0.0028 (13)
C4	0.0385 (15)	0.0387 (18)	0.0251 (14)	−0.0125 (13)	0.0008 (11)	−0.0040 (13)
C5	0.0363 (14)	0.0261 (16)	0.0339 (15)	−0.0072 (11)	0.0089 (13)	−0.0038 (12)
C6	0.0291 (12)	0.0292 (17)	0.0323 (16)	−0.0045 (10)	0.0028 (11)	0.0014 (13)
C7	0.0265 (12)	0.0309 (16)	0.0214 (12)	−0.0037 (11)	0.0010 (11)	−0.0007 (11)
C8	0.0267 (12)	0.0309 (16)	0.0249 (14)	0.0002 (11)	0.0017 (11)	0.0017 (12)
C9	0.0327 (13)	0.049 (2)	0.0299 (15)	−0.0067 (13)	−0.0065 (12)	−0.0059 (13)
C10	0.0533 (18)	0.040 (2)	0.0448 (16)	−0.0017 (14)	0.0079 (18)	−0.0112 (16)
C11	0.0357 (14)	0.0355 (19)	0.0490 (18)	0.0064 (13)	−0.0006 (14)	−0.0014 (15)
C12	0.0318 (14)	0.0416 (18)	0.0288 (15)	0.0003 (12)	−0.0011 (12)	−0.0052 (13)
C13	0.0234 (10)	0.0303 (15)	0.0304 (14)	0.0065 (10)	−0.0005 (12)	−0.0068 (13)
C14	0.0219 (11)	0.0340 (17)	0.0351 (15)	0.0023 (11)	−0.0029 (12)	−0.0059 (13)
C15	0.0238 (12)	0.0369 (18)	0.0369 (15)	0.0039 (11)	−0.0055 (12)	−0.0041 (14)
C16	0.0370 (13)	0.0303 (17)	0.0335 (15)	−0.0001 (12)	−0.0020 (13)	−0.0030 (14)
C17	0.0247 (12)	0.0347 (18)	0.0459 (18)	−0.0028 (12)	−0.0009 (13)	−0.0036 (14)
C18	0.0262 (12)	0.0350 (17)	0.0371 (16)	0.0046 (10)	−0.0061 (14)	−0.0006 (16)

### Geometric parameters (Å, °)

**Table:**

Br1—C16	1.897 (3)	C9—H9C	0.9800
S1—O2	1.491 (2)	C10—H10A	0.9800
S1—C1	1.759 (3)	C10—H10B	0.9800
S1—C13	1.792 (3)	C10—H10C	0.9800
O1—C8	1.365 (3)	C11—H11A	0.9800
O1—C7	1.387 (3)	C11—H11B	0.9800
C1—C8	1.355 (4)	C11—H11C	0.9800
C1—C2	1.453 (4)	C12—H12A	0.9800
C2—C7	1.403 (4)	C12—H12B	0.9800
C2—C3	1.405 (4)	C12—H12C	0.9800
C3—C4	1.385 (4)	C13—C14	1.376 (4)
C3—C9	1.510 (4)	C13—C18	1.397 (3)
C4—C5	1.406 (4)	C14—C15	1.390 (4)
C4—H4	0.9500	C14—H14	0.9500
C5—C6	1.404 (4)	C15—C16	1.381 (4)
C5—C10	1.505 (4)	C15—H15	0.9500
C6—C7	1.377 (4)	C16—C17	1.381 (4)
C6—C11	1.497 (4)	C17—C18	1.369 (5)
C8—C12	1.477 (4)	C17—H17	0.9500
C9—H9A	0.9800	C18—H18	0.9500
C9—H9B	0.9800		
			
O2—S1—C1	109.82 (13)	C5—C10—H10B	109.5
O2—S1—C13	107.25 (12)	H10A—C10—H10B	109.5
C1—S1—C13	99.00 (12)	C5—C10—H10C	109.5
C8—O1—C7	107.00 (19)	H10A—C10—H10C	109.5
C8—C1—C2	108.0 (2)	H10B—C10—H10C	109.5
C8—C1—S1	119.3 (2)	C6—C11—H11A	109.5
C2—C1—S1	132.64 (19)	C6—C11—H11B	109.5
C7—C2—C3	118.0 (3)	H11A—C11—H11B	109.5
C7—C2—C1	103.9 (2)	C6—C11—H11C	109.5
C3—C2—C1	138.1 (2)	H11A—C11—H11C	109.5
C4—C3—C2	116.5 (2)	H11B—C11—H11C	109.5
C4—C3—C9	120.9 (3)	C8—C12—H12A	109.5
C2—C3—C9	122.6 (3)	C8—C12—H12B	109.5
C3—C4—C5	124.3 (3)	H12A—C12—H12B	109.5
C3—C4—H4	117.8	C8—C12—H12C	109.5
C5—C4—H4	117.8	H12A—C12—H12C	109.5
C6—C5—C4	119.8 (3)	H12B—C12—H12C	109.5
C6—C5—C10	120.6 (3)	C14—C13—C18	120.4 (3)
C4—C5—C10	119.6 (3)	C14—C13—S1	119.35 (18)
C7—C6—C5	114.8 (2)	C18—C13—S1	120.0 (2)
C7—C6—C11	121.3 (2)	C13—C14—C15	120.3 (2)
C5—C6—C11	123.9 (2)	C13—C14—H14	119.8
C6—C7—O1	123.1 (2)	C15—C14—H14	119.8
C6—C7—C2	126.6 (2)	C16—C15—C14	118.2 (2)
O1—C7—C2	110.3 (2)	C16—C15—H15	120.9
C1—C8—O1	110.7 (2)	C14—C15—H15	120.9
C1—C8—C12	133.8 (2)	C17—C16—C15	122.0 (3)
O1—C8—C12	115.5 (2)	C17—C16—Br1	119.0 (2)
C3—C9—H9A	109.5	C15—C16—Br1	119.0 (2)
C3—C9—H9B	109.5	C18—C17—C16	119.4 (2)
H9A—C9—H9B	109.5	C18—C17—H17	120.3
C3—C9—H9C	109.5	C16—C17—H17	120.3
H9A—C9—H9C	109.5	C17—C18—C13	119.6 (3)
H9B—C9—H9C	109.5	C17—C18—H18	120.2
C5—C10—H10A	109.5	C13—C18—H18	120.2
			
O2—S1—C1—C8	−137.6 (2)	C8—O1—C7—C2	−0.2 (3)
C13—S1—C1—C8	110.3 (2)	C3—C2—C7—C6	−1.6 (4)
O2—S1—C1—C2	43.6 (3)	C1—C2—C7—C6	178.7 (3)
C13—S1—C1—C2	−68.5 (3)	C3—C2—C7—O1	179.1 (2)
C8—C1—C2—C7	1.1 (3)	C1—C2—C7—O1	−0.5 (3)
S1—C1—C2—C7	−180.0 (2)	C2—C1—C8—O1	−1.3 (3)
C8—C1—C2—C3	−178.5 (3)	S1—C1—C8—O1	179.65 (18)
S1—C1—C2—C3	0.4 (5)	C2—C1—C8—C12	175.0 (3)
C7—C2—C3—C4	−0.3 (4)	S1—C1—C8—C12	−4.1 (4)
C1—C2—C3—C4	179.2 (3)	C7—O1—C8—C1	0.9 (3)
C7—C2—C3—C9	179.5 (2)	C7—O1—C8—C12	−176.1 (2)
C1—C2—C3—C9	−1.0 (5)	O2—S1—C13—C14	13.0 (2)
C2—C3—C4—C5	0.5 (4)	C1—S1—C13—C14	127.2 (2)
C9—C3—C4—C5	−179.3 (3)	O2—S1—C13—C18	−172.7 (2)
C3—C4—C5—C6	1.2 (4)	C1—S1—C13—C18	−58.6 (2)
C3—C4—C5—C10	−179.5 (3)	C18—C13—C14—C15	1.5 (4)
C4—C5—C6—C7	−2.8 (4)	S1—C13—C14—C15	175.7 (2)
C10—C5—C6—C7	177.9 (2)	C13—C14—C15—C16	−1.2 (4)
C4—C5—C6—C11	179.0 (3)	C14—C15—C16—C17	0.6 (4)
C10—C5—C6—C11	−0.3 (4)	C14—C15—C16—Br1	179.5 (2)
C5—C6—C7—O1	−177.7 (2)	C15—C16—C17—C18	−0.2 (4)
C11—C6—C7—O1	0.5 (4)	Br1—C16—C17—C18	−179.2 (2)
C5—C6—C7—C2	3.2 (4)	C16—C17—C18—C13	0.5 (5)
C11—C6—C7—C2	−178.6 (3)	C14—C13—C18—C17	−1.1 (4)
C8—O1—C7—C6	−179.4 (2)	S1—C13—C18—C17	−175.3 (2)

### Hydrogen-bond geometry (Å, °)

**Table:**

Cg1 and Cg2 are centroids of the C2–C7 benzene ring and the C13–C18 bromophenyl ring, respectively.

**Table:**

*D*—H···*A*	*D*—H	H···*A*	*D*···*A*	*D*—H···*A*
C18—H18···O2^i^	0.95	2.58	3.397 (3)	144
C10—H10B···Cg1^ii^	0.98	2.87	3.604 (3)	132
C12—H12B···Cg2^iii^	0.98	2.84	3.671 (3)	143

Symmetry codes: (i) *x*+1/2, −*y*+1/2, *z*; (ii) −*x*+1, −*y*, *z*−1/2; (iii) *x*, *y*, *z*+1.
